# Left and Right Arcuate Fasciculi Are Uniquely Related to Word Reading Skills in Chinese-English Bilingual Children

**DOI:** 10.1162/nol_a_00051

**Published:** 2022-02-10

**Authors:** Yue Gao, Xiangzhi Meng, Zilin Bai, Xin Liu, Manli Zhang, Hehui Li, Guosheng Ding, Li Liu, James R. Booth

**Affiliations:** State Key Laboratory of Cognitive Neuroscience and Learning, and IDG/McGovern Institute for Brain Research, Beijing Normal University, Beijing, China; School of Psychological and Cognitive Sciences, Beijing Key Laboratory of Behavioral and Mental Health, Peking University, Beijing, China; PekingU-PolyU Center for Child Development and Learning, Beijing, China; Max Planck Institute for Psycholinguistics, Nijmegen, The Netherlands; Department of Cognitive Neuroscience and Maastricht Brain Imaging Center, Faculty of Psychology and Neuroscience, Maastricht University, Maastricht, The Netherlands; Department of Psychology and Human Development, Vanderbilt University, Nashville, TN, USA

**Keywords:** arcuate fasciculi, diffusion tensor imaging (DTI), Chinese-English bilingual children, reading skill, phonological awareness, visual spatial ability

## Abstract

Whether reading in different writing systems recruits language-unique or language-universal neural processes is a long-standing debate. Many studies have shown the left arcuate fasciculus (AF) to be involved in phonological and reading processes. In contrast, little is known about the role of the right AF in reading, but some have suggested that it may play a role in visual spatial aspects of reading or the prosodic components of language. The right AF may be more important for reading in Chinese due to its logographic and tonal properties, but this hypothesis has yet to be tested. We recruited a group of Chinese-English bilingual children (8.2 to 12.0 years old) to explore the common and unique relation of reading skill in English and Chinese to fractional anisotropy (FA) in the bilateral AF. We found that both English and Chinese reading skills were positively correlated with FA in the rostral part of the left AF-direct segment. Additionally, English reading skill was positively correlated with FA in the caudal part of the left AF-direct segment, which was also positively correlated with phonological awareness. In contrast, Chinese reading skill was positively correlated with FA in certain segments of the right AF, which was positively correlated with visual spatial ability, but not tone discrimination ability. Our results suggest that there are language universal substrates of reading across languages, but that certain left AF nodes support phonological mechanisms important for reading in English, whereas certain right AF nodes support visual spatial mechanisms important for reading in Chinese.

## INTRODUCTION

There is a long-standing debate about reading across different writing systems, that is, whether reading recruits unique neural resources, constrained by culture (Paulesu et al., 2000; Siok et al., 2004, 2009; M. Zhang, Chen, et al., 2014), or whether reading involves universal neural processes, independent of language (Frost, 2012; Rueckl et al., 2015). Studies that have researched this question have been based on functional imaging studies examining neural activation. Skilled reading not only requires engagement of multiple cortical regions, but also adequate structural connection between areas (Ben-Shachar et al., 2007; see reviews, Vandermosten, Boets, Wouters, & Ghesquière, 2012; Xiaosha Wang et al., 2012; M. Zhang, Li, et al., 2014).

A word’s pronunciation is generated via propagating activation from processing units representing orthographic input along connections to other units engaged in phonological output. Connectionist models in alphabetic languages argue that early reading relies largely on this orthography-to-phonology component (Harm & Seidenberg, 2004; Joanisse & McClelland, 2015; Seidenberg & McClelland, 1989). An adapted connectionist model for Chinese reading suggests a different developmental pattern, such that mappings from orthography-to-phonology are not as important in early reading (J. Yang et al., 2009). This language difference in early reading might be due to the language specific properties of English versus Chinese. English words are composed of letters organized from left-to-right, and word reading follows a unidirectional scanning path. English words are predominantly read out by mapping printed letters onto phonemes, and therefore phonological awareness is an essential skill (Bradley & Bryant, 1983; Ramus, 2014; Shaywitz & Shaywitz, 2005; Snowling, 2001; Wagner & Torgesen, 1987).

In contrast, Chinese is usually described as a *logographic* language. Chinese characters are square configurations packed with intricate strokes, occupying a two-dimensional space both in height and width. A stroke is an unbroken line drawn in a single movement, and it is the smallest single component of a Chinese character (McBride-Chang, 2016). Characters are composed of strokes in different directions, possessing varying geometric properties. For example, characters can be arranged from left to right (部, unit), from top to bottom (杏, plum), or enclosed (田, field). These properties can be rearranged to make up new characters, for example, 部 (unit) versus 陪 (to accompany); 杏 (plum) versus 呆 (to stay); 田 (field) versus 叶 (leaf). Studies have found that better visual spatial ability is related to higher Chinese reading skill (Ho & Bryant, 1999), and that configural knowledge of visual spatial components of Chinese characters predicts reading skill in both kindergarten and primary grades (Luo et al., 2013).

White matter fiber bundles play an essential role in the fast transmission of neural signals between different cortical regions (Friederici, 2015; Wake et al., 2011). One important language-related white matter tract is the *arcuate fasciculus* (AF), which originates from the left posterior superior temporal gyrus, runs to the inferior parietal lobule, and then further connects to the inferior frontal area (Catani et al., 2005). Because this tract directly connects the receptive and expressive processing of phonemes during speech, the left AF is argued to subserve the dorsal phonological route, which might be particularly important during the reading acquisition of children (Glasser & Rilling, 2008). In alphabetic languages, the *fractional anisotropy* (FA) of the left AF has been shown to be correlated with word reading skills (Huber et al., 2018; see the review in Vandermosten, Boets, Wouters, & Ghesquière, 2012; Yeatman et al., 2012); to be different between typical and atypical prereaders or early readers (Myers et al., 2014; Saygin et al., 2013; Xiaojuan Wang et al., 2016); to be different between infants with and without risk for developmental dyslexia (Langer et al., 2017); and to predict longitudinal reading gains (Gullick & Booth, 2015). The FA of the left AF is also consistently correlated with children’s phonological awareness (Saygin et al., 2013; Vandermosten et al., 2012; Yeatman et al., 2011), indicating that phonology may mediate the relationship between the left AF and alphabetic reading skills. The left AF is part of the dorsal temporo-parieto-frontal pathway, which involves posterior portions of the superior temporal gyrus, supramarginal gyrus, and inferior frontal gyrus. These regions are consistently found to be active during phonology-related tasks (Fiez & Petersen, 1998; Gandour et al., 2002; Temple, 2002), and they are more involved in alphabetic reading compared to Chinese reading (Bolger et al., 2005; Tan et al., 2005), so the dorsal pathway is proposed to be a phonology-mediated route (Jobard et al., 2003; Schlaggar & McCandliss, 2007).

In contrast to alphabetic scripts, few studies (Fan et al., 2020; Su et al., 2017, 2018, 2020; H. L. S. Wang et al., 2019) have examined how structural connectivity supports non-alphabetic reading. Among these studies, three (Su et al., 2017, 2018, 2020) reported findings on the left AF. One study reported that children with consistently poor oral vocabulary development rate tend to show a significantly reduced FA in the posterior and direct segments of the left AF and this vocabulary development rate can predict the FA of the left AF (Su et al., 2017). Further, the same group reported that the FA of the direct segment of the left AF is different between typical and atypical Chinese readers, and that it is positively correlated with phonological awareness ability (Su et al., 2018). In addition, age of literacy exposure is found to be correlated with fractional anisotropy of the direct segment of the left AF (Su et al., 2020). These results suggest that Chinese reading might share mechanisms along the left AF pathway with alphabetic reading. However, since Chinese is a tonal language, Chinese reading may need additional support from the right hemisphere. Lexical tones are a feature of the Chinese language. There are four contrastive tones in Chinese, which can be distinguished by the shapes of the internal pitch contour: Tone 1 is high-level; Tone 2 is high-rising; Tone 3 is low-dipping; and Tone 4 is high-falling. Tone awareness has been shown to explain variability in Chinese reading skill in kindergarten children (Shu et al., 2008) and in third graders (Yin et al., 2011). Functional neuroimaging studies have implicated the right hemisphere in processing pitch-related acoustic information (Qi et al., 2015; see the review in Wong, 2002). Structural connectivity studies have also suggested that the right AF may play a role in the prosodic processing of language (Sammler et al., 2015). The anterior segment of the right AF, which connects the right inferior parietal lobule and the inferior frontal cortex, has been found to be positively correlated with Chinese learning scores (Qi et al., 2015). In this study, Chinese learning scores consisted of speech, listening, and reading, so the researchers speculate that better learning might reflect sensitivity to the tonal properties of the Chinese language.

In addition to the demand on tonal information for processing spoken words, the square-shaped graphic form of Chinese characters demands intensive processing (Tan, 2005), so Chinese reading might also need greater engagement of visual spatial ability supported by the right hemisphere. Several studies have suggested the right parietal lobule and the right intraparietal sulcus are important for Chinese reading. Abnormal activation of the right inferior parietal sulcus was found in Chinese dyslexia (Siok et al., 2004). Intrinsic connectivity between the right parietal lobule and the right middle occipital gyrus was also found to be positively correlated with Chinese reading skill (Wang et al., 2012). With the evidence postulating the importance of the right AF in visual spatial processing (Doricchi et al., 2008; Hoeft et al., 2007; Makris et al., 2005; Rauschecker et al., 2009; Thiebaut de Schotten et al., 2014), and the fact that visual spatial processing is demanded by the high-density characters in Chinese reading (L. Liu et al., 2012; Nelson et al., 2009; Perfetti et al., 2006; Tan et al., 2005), we hypothesize that reading in Chinese should be uniquely supported by visual spatial processing in the right AF pathway.

### The Current Study

In our study, we tracked the three segments of the bilateral AF based on the model proposed by Catani et al. (2005). The direct pathway connects the posterior part of both superior (BA22) and middle (BA21) temporal cortex with the inferior frontal cortex (BA 44 and 45). The anterior part connects the inferior parietal cortex (BA39 and 40) with inferior frontal cortex. The posterior part connects temporal cortex with inferior parietal cortex. We implemented a developed algorithm, Automated Fiber Quantification (Yeatman et al., 2012), which divides the segment between any two regions of interest (ROIs) into many nodes and calculates each node’s FA to improve the anatomical specificity, tackling the limits from whole-tract methods. Large bundles like the AF include many fasciculi, but not all fasciculi traverse the full length of the tract (Wandell, 2016; Yeatman et al., 2012). Because of this, important structural information may be reflected only at specific locations along the fasciculi.

We explored common and unique relationships of the tissue properties of bilateral AF with reading skill in English and Chinese. Our research employed a within-subject design. We recruited as participants Chinese-English bilingual children who started learning to read in Chinese and English approximately at the same age (6 years old). Although we expected some overlap in the correlation of reading skill across languages with the tissue properties of the AF, we predicted that the left AF would be uniquely correlated with English reading skill, whereas the right AF would be uniquely associated with Chinese reading skill. Among the nodes that showed significant correlation with reading skill, we expected that English-reading nodes would be correlated with an independent measure of phonological awareness ability, whereas the Chinese-reading nodes would be correlated with an independent measure of visual spatial ability.

## MATERIALS AND METHOD

### Participants

A total of 40 participants (25 males and 15 females; age: 8.2–12.0 years old) from primary schools in Beijing were recruited for the scanning and behavioral assessment. The participants’ home language and social language was Chinese, but all participants were Chinese-English bilinguals who started learning to read both Chinese and English at approximately 6 years old and received about four English classes and four Chinese classes per week (45 minutes per class). In our study, *Chinese-English bilinguals* refers to children who learned Chinese as their first language (L1) and English as their second language (L2) and began to receive formal Chinese and English literacy instruction at around age 6 (Chow, 2014; Gao et al., 2019). The children’s L1 Chinese was more dominant than their L2 English because exposure to their L1 was more frequent compared to their L2 (Siu & Ho, 2020).

All children were typically developing without any psychiatric or neurological disorders as reported by their parents. None of them had reading difficulty in Chinese or English, based on the report of their teachers. They were all right-handed according to self-report (Edinburgh Handedness Inventory; Oldfield, 1971), with normal hearing, and normal or corrected-to-normal vision. Informed consent was obtained from each participant and their parents. The Institutional Review Board of Beijing Normal University Imaging Center for Brain Research approved the protocol.

### Behavioral Assessment

***Reading skill***: English reading skill was assessed with Word Identification, a subtest from the Woodcock Johnson Assessment Test-Revised (Woodcock, 1987). Children were asked to read aloud English letters and words. There was a total of 53 words, and the test stopped when the child failed to recognize 6 consecutive words. Raw scores of this test were used due to the lack of Chinese norms for the Word Identification test. Chinese reading skill was measured by the Character Identification Test. This test included 150 characters with increasing difficulty, and children were required to read them aloud. Testing stopped when the child failed to recognize 10 consecutive characters. This test is widely used for examining reading skill in Chinese children (Li et al., 2012; You et al., 2011). We also used raw scores in this test to parallel the use of raw scores in the English reading test.
***Phonological awareness ability***: English phonological awareness was measured with onset phoneme deletion, in which children were asked to pronounce an item after the initial phoneme of the syllable was removed (You et al., 2011). Chinese phonological awareness was also assessed using phoneme deletion (M. L. Zhang et al., 2018). For example, /Mei/ without the initial sound would be /ei/. For each language, there was a total of 20 items, and the test was stopped when the child failed on 5 consecutive items. Raw scores were used.
***Visual spatial ability***: We administered the Block Design subtest of the Wechsler Intelligence Scale for Children—Third Edition (WISC-III, Wechsler, 1991). This task required participants to recreate various two-dimensional geometric patterns with cubes in a specific time period. Administration and scoring followed the instructions in the testing manual of the Chinese version. Raw scores were used.
***Chinese tone discrimination ability***: For each item, children heard 4 characters that were read aloud by the examiner. Among the 4 characters, 3 of them shared the same tone. Children were asked to select the character that had a different tone from the others. There was a total of 20 items (Meng et al., 2011). Raw scores were used.
***Nonverbal Intelligence (IQ)***: Raven’s standard progressive matrices (Raven & Court, 1998) was used. Participants were asked to select a plate from six to eight alternatives to complete a visual matrix. Participants were scored based on the Chinese norms.

The behavioral performance of the participants is shown in Table 1. Participants with a range of reading skills were recruited to provide variability for the brain-behavior correlation analysis. All the participants had an IQ above average based on the Raven’s standard progressive matrices.

**Table 1.** Behavioral performance of the participants

	Mean (*SD*)	Range
English word identification	16.6 (4.6)	6–24
Chinese character identification	111.1 (17.2)	70–140
English phonological awareness	5.6 (2.1)	1–8
Chinese phonological awareness^a^	13.9 (4.1)	3–19
Visual spatial ability^b^	43.9 (12.3)	21–79
Chinese tone discrimination	13.9 (4.3)	5–20
Raven’s standard progressive matrices^c^	75.9 (15.4)	50–95

*Note*. All the scores in the table are raw scores except for the Raven’s. *SD* = standard deviation.

^a^ One participant lacked Chinese phonological awareness.

^b^ Four participants lacked visual spatial ability.

^c^ Percentiles.

### Behavioral Data Analysis

To explore the contribution of phonological awareness and visual spatial ability to English or Chinese reading skill separately, hierarchical stepwise regressions were performed in SPSS 26.0 with age and sex as covariates.

### Data Acquisition

All 40 participants underwent MRI scanning on a 3T system (Siemens Trio Tim), with a 32-channel head coil. The diffusion tensor imaging (DTI) data were acquired using an echoplanar imaging (EPI) sequence (echo time [TE] = 89 ms; repetition time [TR] = 8,000 ms; field of view [FOV] = 282 × 282 mm; matrix size = 128 × 128; phase encoding [PE] direction = anterior − posterior; phase partial Fourier = 6/8). Parallel acquisition was conducted in the GRAPPA mode, with reference line PE  =  38, and an acceleration factor of 2 for all the images. Each 2 mm thick, 30 diffusion-weighted volumes (b = 1,000 s/mm^2^) and one reference volume (b = 0 s/mm^2^) were acquired using a standard diffusion direction matrix. The measurements were repeated twice to enhance the signal-to-noise ratio.

T1-weighted images were acquired using magnetization prepared rapid gradient echo (MPRAGE) sequence, with the following parameters: TR = 2,300 ms, TE = 4.18 ms, flip angle = 9°, FOV = 256 × 256 mm, matrix size = 256 × 256, slice thickness = 1 mm, and voxel size = 1.0 mm × 1.0 mm × 1.0 mm.

### DTI Data Quality Assurance

We implemented a standard pipeline developed by Lauzon et al. (2013). By incorporating multiple statistical metrics that evaluate the collected image data, the processed tensor parameters, together with the output from the artifact detection software package, DTIPrep (https://www.nitrc.org/projects/dtiprep/), this pipeline produced quality assurance reports for each subject. The following metrics were evaluated: head motion, bad slices, voxel outlier detection, noise-sensitive evaluation of fitting errors, signal-to-noise ratio level, and quality of tensor fit. Based on the report, visual inspection of the data was conducted to detect potential artifacts. Three subjects’ data were excluded due to poor data quality. For the head motion, we calculated the Euclidean distance of the transformation (*x*, *y*, *z*).

### DTI Data Analysis

Data preprocessing was conducted using *mrDiffusion*. This is an open-source package (https://web.stanford.edu/group/vista/cgi-bin/wiki/index.php/MrDiffusion). First, T1-weighted images were aligned to the anterior commissure-posterior commissure (AC-PC) orientation. Diffusion weighted images were corrected for eddy-current distortions and head motion (Rohde et al., 2004). Diffusion weighted volumes were registered to the non-diffusion weighted (b0) volume, which was registered to the T1-weighted image using a rigid body mutual information maximization algorithm (implemented in SPM8; Ashburner & Friston, 2007). Then, the combined transform resulting from motion correction, eddy-current correction, and anatomical alignment was applied to the raw diffusion data once. Next, the table of gradient directions was appropriately adjusted to fit the resampled diffusion data (Leemans & Jones, 2009). The raw diffusion data was then fitted with the tensor model using a standard least-squares algorithm. Fractional anisotropy was calculated as the normalized standard deviation of the eigenvalues (λ1, λ2, λ3). We focused specifically on the FA metric because it is a robust measure of the degree of anisotropic diffusion occurring within a voxel. In addition, it has been of primary interest in the previous studies exploring the relationship between white matter microstructural properties and reading skills across languages (Gullick & Booth, 2015; Qi et al., 2015; Su et al., 2020; Vanderauwera et al., 2015; for reviews, see Vandermosten, Boets, Wouters, & Ghesquière, 2012).

### Automated Tractography Procedure

After preprocessing, the corrected DTI maps were submitted to the tractography algorithm. Deterministic whole-brain streamline tractography was performed using streamlines tractography (STT) algorithm in Automated Fiber Quantification (AFQ) with a fourth-order Runge-Kutta path integration method and 1-mm fixed-step size (Basser et al., 2000; Mori et al., 1999). A continuous tensor field across the brain was estimated, and whole-brain fiber tracking was initialized from seed points within a white matter mask containing all voxels where FA was >0.3 and proceeded in both directions along the principal diffusion axis. Tracking was terminated if FA was below the threshold 0.2 and the fiber angle changed >30°. These tracking thresholds were adopted in previous studies using child participants (Goodrich-Hunsaker et al., 2018; Yeatman et al., 2012). This tracking step generated a candidate database of fibers for the whole brain that were parcellated to identify anatomically defined fasciculi. Fiber tracts that pass through two waypoint ROIs (Wakana et al., 2007) were assigned to a particular fiber group. The direct, anterior, and posterior segments of the AF were examined. The direct segment was identified as the AF by AFQ. The anterior segment corresponded best to the superior longitudinal fasciculus (SLF) by AFQ, connecting the frontal lobe and the inferior parietal lobule. For the posterior segment, the AFQ segmentation procedure was modified to include this additional fiber tract. (See detailed description in Supplementary Information; Supporting Information can be found at https://doi.org/10.1162/nol_a_00051.) Three tracts (direct, anterior, and posterior AF) are depicted on a representative participant (Figure S1).

ROIs were defined in Montreal Neurological Institute (MNI) space using the Mori atlas (Mori et al., 2008). Fiber tract refinement was accomplished by comparing each candidate fiber to the fiber tract probability map created by Hua et al. (2008). Candidate fibers tracked through regions of low probability were discarded, and the shape of the tracts was defined. Due to noise in the data, complex fiber orientation, and other confounds, one extra procedure was implemented to clean a few fibers that were substantially different from other fibers in the group. AFQ calculated the fiber’s distance from the core of the fiber tract and then removed the fibers that were more than 5 standard deviations from the core or 4 standard deviations above the mean fiber length until no outliers were detected. Therefore, the remaining fibers were coherently bundled together, branching toward their common destination and defined as one fasciculus. The fasciculi were clipped to one central portion, spanning between the two waypoints ROIs. The central portion (direct and anterior segments) was then resampled to 100 equally spaced nodes. (For the posterior segment, due to a relatively shorter fiber length, it was resampled to 30 equivalent nodes.) The FA value was calculated and summarized at each node by taking a weighted average. We further checked the realignment and inspected the cleaned tracts for anatomical correctness. Taken together, out of 37 subjects, 35 (95%) left direct AF, 30 (81%) right direct AF; 36 (97%) left anterior AF, 30 (81%) right anterior AF; 37 (100%) left posterior AF, 37 (100%) right posterior AF were tracked appropriately.

### Statistical Analyses

After we obtained the mean and standard deviation of the FA value at each node, we used Pearson correlation to elucidate the relationships between reading skill and point-wise FA values along the three segments of the bilateral AF (controlling for age and sex). The correlation between white matter tissue properties and behavior tests has been investigated in many previous reading-related studies (Saygin et al., 2013; Su et al., 2018, 2020; Vandermosten et al., 2012; Yeatman et al., 2011). As phonological awareness is thought to be critical for English reading (Bradley & Bryant, 1983; Ramus, 2014; Shaywitz & Shaywitz, 2005; Snowling, 2001; Wagner & Torgesen, 1987), while visual spatial or Chinese tone discrimination ability is thought to be critical for Chinese reading (Shu et al., 2008; Yin et al., 2011), we further used Pearson correlation to elucidate the relationships between these cognitive abilities and FA values of the nodes showing significant correlation with reading skills.

Here we describe the analysis procedure using the direct segment as an example. First, we performed a conjunction analysis, in which nodes that were correlated with both Chinese and English skills, after controlling for age and sex, were defined as common variables. To find the nodes that were unique to English reading skill, we controlled for Chinese reading skill, age, and sex, and then calculated the partial correlation between English reading skill and the FA value at each node. Similarly, to find nodes that were unique to Chinese reading skill, we controlled for English reading skill, age, and sex, and then conducted partial correlation analysis between Chinese reading skill and the FA value at each node. Finally, in these significant language-common or language-specific clusters, we further examined whether the mean FA of these clusters was associated with cognitive abilities using Pearson’s correlation in the Robust correlation toolbox (Pernet et al., 2013). Partialling for age and sex, FA values of these significant “English nodes,” “Chinese nodes,” and “Common nodes” were correlated with cognitive abilities to determine whether these regions were related to phonological awareness, visual spatial ability, or Chinese tone discrimination ability. We did not control for head motion as no significant correlation was found between head motion and FA values or reading skills. Fisher’s *z* test was used to compare correlation coefficients between mean cluster FA and different cognitive abilities, in order to discover whether one cognitive ability was more highly correlated with a mean cluster FA compared to another cognitive ability.

Given the high degree of correlation between neighboring points on the tract, nodes should not be treated as independent variables. Thus, the Bonferroni correction was too conservative. To control for multiple comparisons, we performed an implementation of the permutation method described by Nichols and Holmes (2002) and used in several studies (David et al., 2020; Yeatman et al., 2012). By modifying and running the function AFQ_MultiCompCorrection to correct partial correlation results, the data were randomly shuffled for 1,000 permutations and a distribution of “chance” correlations for every correlation that we ran was created. We then created a final distribution from the maximum cluster size of these permutations, considering all of the correlations, and compared the actual (nonshuffled) cluster size with these values to assign the significance alpha and cluster threshold at *p* < 0.05 (two-tailed). This returned a family-wise error (FWE) corrected cluster size, which meant that significant clusters of this size or greater pass the multiple comparison threshold and did not need further *p* value adjustment (Dodson et al., 2017; Dubner et al., 2020; Travis et al., 2015, 2016, 2017). Some previous studies also have adopted lenient criteria (Banfi et al., 2019; Dodson et al., 2017), for example, with more than 3 of adjacent nodes under the quantification of the diffusion metrics along each fiber tract at 30 equidistant nodes (Dodson et al., 2017). Thus, in our study, results were reported (1) at a stringent threshold, requiring a sufficient number of adjacent nodes to meet the criteria for a FWE corrected cluster size, or (2) at a more lenient threshold, requiring ≥9 adjacent nodes along FA tract profiles resampled to 100 equally spaced nodes or ≥3 adjacent nodes along FA tract profiles resampled to 30 equally spaced nodes, both at *p* < 0.05 uncorrected.

## RESULTS

### Behavioral Results

To explore the contribution of phonological awareness and visual spatial ability to English or Chinese reading skill separately, hierarchical stepwise regressions were performed. Results revealed that Chinese phonological awareness (*R*^2^ change = 0.081) and visual spatial ability (*R*^2^ change = 0.066) contributed significantly and uniquely to Chinese reading ability after controlling for age (Table 2). Meanwhile, English phonological awareness (*R*^2^ change = 0.227) contributed significantly to English reading ability after controlling for age (Table 3). Sex failed to be a significant predictor for Chinese or English reading skill, and visual spatial ability failed to be a significant predictor for English reading skill.

**Table 2.** Cognitive abilities predicting Chinese reading skill using hierarchical stepwise regression analysis

Variables	β	*t*	*R* ^2^	*R*^2^ change	*F*
Step1					
Age	0.633	4.768***	0.401	0.401	22.733***
Step2					
Age	0.568	4.414***	0.482	0.081	15.347***
Chinese PA	0.292	2.274*			
Step3					
Age	0.450	3.375**	0.548	0.066	12.952***
Chinese PA	0.343	2.761**			
Visual spatial ability	0.283	2.170*			

*Note*. This table shows the results of stepwise hierarchical regression analysis with age, sex, Chinese phonological awareness, and visual spatial ability as the independent variables and Chinese reading skill as the dependent variable. Results show that sex failed to be a significant predictor for Chinese reading skill. Age entered into the regression model at the first step, with Chinese phonological awareness at the second step, and visual spatial ability at the third step. It was found that both Chinese phonological awareness and visual spatial ability had significant unique contributions to Chinese reading skill after age was controlled for.

*p < 0.05, **p < 0.01, ***p < 0.001; PA, phonological awareness.

**Table 3.** Cognitive abilities predicting English reading skill using hierarchical stepwise regression analysis

Variables	β	*t*	*R* ^2^	*R*^2^ change	*F*
Step1					
Age	0.426	2.868**	0.183	0.183	8.226**
Step2					
Age	0.276	2.060*	0.410	0.227	12.493***
English PA	0.500	3.727***			

*Note*. This table shows the results of stepwise hierarchical regression analysis with age, sex, English phonological awareness, and visual spatial ability as the independent variables and English reading skill as the dependent variable. Results show that sex and visual spatial skill failed to be a significant predictor for English reading skill. Age entered into the regression model at the first step, with English phonological awareness at the second step. It was found that only English phonological awareness had significant unique contributions to English reading skill after age was controlled for.

*p < 0.05, **p < 0.01, ***p < 0.001; PA, phonological awareness.

### DTI Results

**Figure 1.**
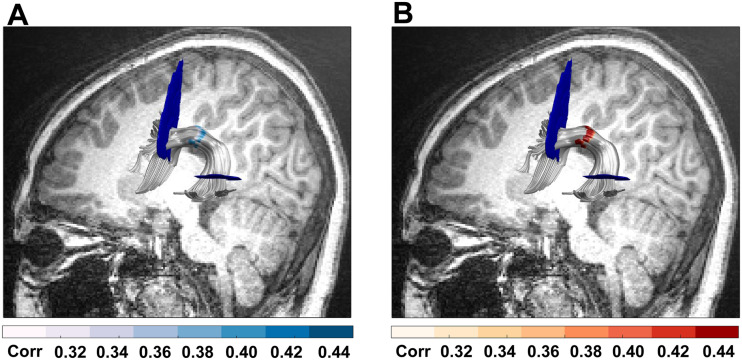
Nodes of the direct segment of the left arcuate fasciculus (AF) common to Chinese and English reading skills. Common nodes (nodes 37 to 45) along the direct segment of the left AF, whose fractional anisotropy values were positively correlated with both (A) English reading skill (blue) and (B) Chinese reading skill (orange). Age and sex were controlled in (A) and (B). These results passed the lenient threshold for adjacent nodes ≥9 at *p* < 0.05 uncorrected, but did not survive a stringent threshold of FWE cluster size correction for adjacent nodes ≥16, *p* < 0.05).

To discover common segments for English and Chinese reading, conjunction analysis was performed. The distribution of the correlation coefficients between FA of the left AF direct nodes and reading skill in Chinese and English is shown in Figure S2. We found that along the left direct segment AF, near the temporoparietal area, FA values from node 37 to node 45 were associated with reading skill in both Chinese and English partialling for age and sex. (These results passed the lenient threshold for adjacent nodes ≥9 at *p* < 0.05 uncorrected, but neither English nor Chinese results survived the stringent threshold of FWE cluster size correction for adjacent nodes ≥16, *p* < 0.05; Figure 1.) These nodes were not correlated with phonological awareness, visual spatial ability, or Chinese tone discrimination ability. We found no overlapping correlations between languages in other nodes in the left hemisphere or in the right AF.

**Figure 2.**
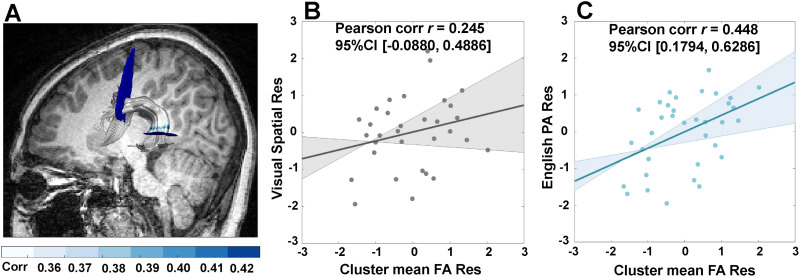
Nodes of the direct segment of the left arcuate fasciculus (AF) specific to English reading skill. (A) English-unique reading nodes (node 80 to node 95; survived a stringent threshold of FWE cluster size correction for adjacent nodes ≥15, *p* < 0.05) along the direct segment of the left AF, whose fractional anisotropy (FA) values were positively correlated with English reading skill (blue) (partialling for Chinese reading skill, age, and sex). (B) No significant correlation between visual spatial ability and the mean FA of the English reading nodes, partialling for age and sex. Thirty-two subjects’ data were used in this correlation as three subjects lacked visual spatial scores among the 35 subjects with the direct segment of the left AF successfully tracked. (C) Significant positive correlation between English phonological awareness (PA) ability and the mean FA of the English reading nodes, partialling for age and sex. The shadows in (B) and (C) represent 95% bootstrapped confidence intervals and the straight lines refer to the regression. Res = residual.

To find the nodes unique to English reading skill, partial correlations were calculated. The distribution of the correlation coefficients between FA of the left AF direct nodes and English reading skill is shown in Figure S3. Along the direct segment of the left AF, we found that near the superior temporal cortex, FA values from node 80 to node 95 were significantly correlated with English reading skill when Chinese reading skill, sex, and age were partialled out (survived the stringent threshold of FWE cluster size correction for adjacent nodes ≥15, *p* < 0.05; Figure 2A). The mean FA of the cluster in nodes 80–95 specific to English reading skill was further positively correlated with English phonological awareness ability (*r* = 0.448, *p* = 0.009; Figure 2C and Table S1), but not with visual spatial ability (*r* = 0.245, *p* = 0.193; Figure 2B and Table S1). However, a Fisher *z* test did not find significant differences between the correlations of FA-phonological awareness ability and FA-visual spatial ability (*z* = 0.91, *p*_2-tailed_ = 0.36). We did not find any nodes specific to English reading skill in other segments in the left AF or in the right AF (Table S4).

**Figure 3.**
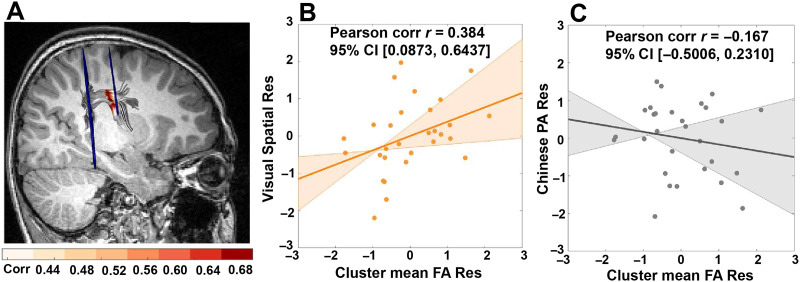
Nodes of the anterior segment of the right arcuate fasciculus (AF) specific to Chinese reading skill. (A) Chinese-unique reading nodes (node 8 to node 48; survived the stringent threshold of FWE cluster size correction for adjacent nodes ≥24, *p* < 0.05) along the anterior segment of the right AF, whose fractional anisotropy (FA) values were positively correlated with Chinese reading skill (orange) (partialling for English reading skill, age, and sex). (B) Significant positive correlation between visual spatial ability and the mean FA of Chinese-unique reading nodes, partialling for age and sex. (C) Not significant correlation between Chinese phonological awareness (PA) ability and the mean FA of Chinese-unique reading nodes, partialling for age and sex. The shadows in (B) and (C) represent 95% bootstrapped confidence intervals and the straight lines refer to the regression. Note: One subject was excluded from the correlation with the cognitive tests due to lack of visual spatial and Chinese PA scores among the 30 subjects with anterior segment of the right AF successfully tracked. Res = residual.

To find the nodes unique to Chinese reading skill, partial correlations were calculated. Figure 3A illustrates that along the anterior segment of the right AF, nodes 8 to 48 showed a positive correlation between the FA values and Chinese reading skill when controlling for English reading skill, sex, and age (survived in stringent threshold of FWE cluster size correction for adjacent nodes ≥24, *p* < 0.05), but not with English reading skill. Further, the mean FA of this cluster demonstrated a positive correlation with visual spatial ability (*r* = 0.384, *p* = 0.048; Figure 3B and Table S2), but not with Chinese phonological awareness ability (*r* = −0.167, *p* = 0.405; Figure 3C and Table S2), or with Chinese tone discrimination ability (*r* = −0.260, *p* = 0.191; Table S2). The Fisher *z* test found that there was a significant difference between the correlations of the FA-visual spatial ability and FA-Chinese phonological awareness ability (*z* = 2.07, *p*_2-tailed_ = 0.039), and a significant difference between the correlation of FA-visual spatial ability and FA-Chinese tone discrimination ability (*z* = 2.42, *p*_2-tailed_ = 0.016). The distribution of the correlation coefficients between FA of right AF anterior nodes and Chinese reading is shown in Figure S4.

**Figure 4.**
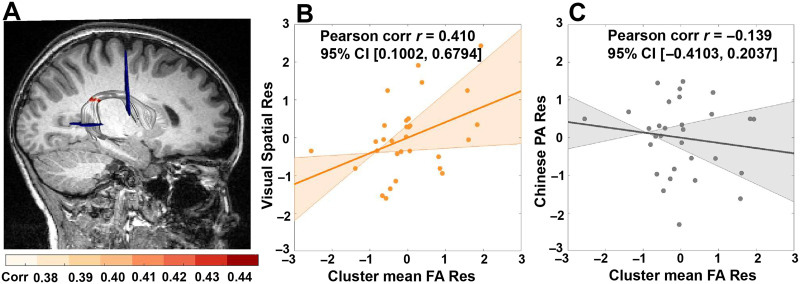
Nodes of the direct segment of the right arcuate fasciculus (AF) specific to Chinese reading skill. (A) Chinese-unique reading nodes (node 53 to node 61; passed the lenient threshold for adjacent nodes ≥9 at *p* < 0.05 uncorrected, but did not survive the stringent threshold of FWE cluster size correction for adjacent nodes ≥15, *p* < 0.05) along the direct segment of the right AF, whose fractional anisotropy (FA) values were positively correlated with Chinese reading skill (orange) (partialling for English reading skill, age, and sex). (B) Significant positive correlation between visual spatial ability and the mean FA of Chinese-unique reading nodes, partialling for age and sex. (C) No significant correlation between Chinese phonological awareness (PA) ability and the mean FA of Chinese-unique reading nodes, partialling for age and sex. The shadows in (B) and (C) represent 95% bootstrapped confidence intervals and the straight lines refer to regression lines. Res = residual.

The distribution of the correlation coefficients between the FA of the nodes in the right AF direct segment and Chinese reading skill is shown in Figure S5. Two clusters were correlated uniquely with Chinese reading skill. First, as shown in Figure 4A, near the temporoparietal region in the direct segment of the right AF, higher FA values from node 53 to node 61 correlated with Chinese reading skill when controlling for English reading skill, sex, and age. (These results passed the lenient threshold for adjacent nodes ≥9 at *p* < 0.05 uncorrected, but did not survive the stringent threshold of FWE cluster size correction for adjacent nodes ≥15, *p* < 0.05.) Further, the mean FA of this cluster showed a significantly positive correlation with visual spatial ability (*r* = 0.410, *p* = 0.030; Figure 4B and Table S3), but not with Chinese phonological awareness ability (*r* = −0.139, *p* = 0.482; Figure 4C and Table S3), or with Chinese tone discrimination ability (*r* = 0.028, *p* = 0.887; Table S3). Furthermore, the Fisher *z* test detected significant differences between the correlations of FA-visual spatial ability and FA-Chinese phonological awareness ability (*z* = 2.11, *p*_2-tailed_ = 0.035), but no significant difference between the correlations of the FA-visual spatial ability and the FA-tone discrimination ability (*z* = 1.5, *p*_2-tailed_ = 0.134). Second, the FA values from node 10 to node 18, near the premotor region, were also significantly correlated with Chinese reading skill, partialling for English reading skill, sex, and age. (These results passed the lenient threshold for adjacent nodes ≥9 at *p* < 0.05 uncorrected, but failed to pass the stringent threshold of FWE cluster size correction for adjacent nodes ≥15, *p* < 0.05.) However, the mean FA of this cluster (nodes 10–18) was not correlated with visual spatial ability (*r* = 0.328, *p* = 0.089), Chinese phonological awareness (*r* = −0.079, *p* = 0.691), or Chinese tone discrimination (*r* = −0.084, *p* = 0.671).

The distribution of the correlation coefficients between the FA of the nodes in the left AF posterior segment and Chinese reading skill is shown in Figure S6. Along the posterior segment of the right AF, near the superior temporal gyrus, nodes 6 to 10 showed negative correlations between FA values and Chinese reading skill when controlling for English reading skill, sex, and age (results passed the lenient threshold for adjacent nodes ≥3, but failed to pass the stringent threshold of FWE cluster size correction for adjacent nodes ≥7, *p* < 0.05), but not with English reading skill. However, the mean FA of this cluster (nodes 6–10) was not correlated with visual spatial ability (*r* = 0.206, *p* = 0.259), Chinese phonological awareness (*r* = 0.104, *p* = 0.551), or Chinese tone discrimination (*r* = 0.155, *p* = 0.374). We did not find any significant results specific to Chinese reading skill along the posterior segment in the right AF or anterior segment in the left AF (Table S4).

## DISCUSSION

The goal of this study was to investigate shared and unique white matter substrates for reading skill across languages in a group of Chinese-English bilingual children. We tracked the three segments of bilateral arcuate fasciculi and correlated fractional anisotropy along these segments with reading skill, and its underlying cognitive components. There were four main findings. First, behavioral data showed that phonological awareness significantly contributed to both English and Chinese reading, while visual spatial ability uniquely contributed to Chinese reading skill. Second, better reading skill in both English and Chinese was correlated with higher FA in the rostral part of the direct segment of the left AF. Third, better reading skill only in English was correlated with higher FA in the caudal part of the direct segment of the left AF, and this part was correlated with phonological awareness ability. Fourth, better reading skill only in Chinese was correlated with higher FA in the direct and anterior segments of the right AF, and these parts were correlated with higher visual spatial ability. These results suggest that there are both shared and unique neural substrates for reading in English and Chinese that depend on the cognitive demands of the writing system.

### Shared Between Writing Systems

We found that better reading skill in both English and Chinese was correlated with the direct segment of the left AF in the frontoparietal region, suggesting that these two strikingly different languages share certain common neural support. FA values of the frontoparietal region were not correlated with phonological awareness or visual spatial ability, indicating that the correlation with reading skill may be due to some general mechanisms involved in mapping from visual symbols to speech sounds. Our finding is consistent with one tract-based spatial statistics (TBSS) study (Zhang, Chen, et al., 2014) that reported that FA values in the frontoparietal region were correlated with both Chinese and English reading skills. Although few studies have reported the association between Chinese reading and white matter metrics, many studies have found associations for English reading. Studies using the voxel-based method in alphabetic scripts have shown that lower FA values in the left temporoparietal region are correlated with poorer word reading skill (Beaulieu et al., 2005; Nagy et al., 2004; Odegard et al., 2009), poorer pseudoword reading skill (Klingberg et al., 2000; Odegard et al., 2009; Steinbrink et al., 2008), and slower reading fluency (Gold et al., 2007). This region tends to be correlated with phonological awareness ability (Deutsch et al., 2005; see reviews in Vandermosten, Boets, Wouters, & Ghesquière, 2012), again suggesting a general role for this white matter segment in mapping between orthography and phonology.

### Unique to English Reading

Our study also found that along the direct segment of the left AF, higher FA values in the caudal nodes near the superior temporal gyrus were uniquely correlated with better reading skill in English, and these nodes were also associated with better phonological awareness ability in that language. Phonological awareness was measured with a phoneme deletion task that required the segmentation of a sound from a word, and the pronunciation of the remaining parts of the word. The left AF might support a dorsal phonological-articulatory route (Glasser & Rilling, 2008), in which speech information is decoded phonologically in the posterior superior temporal gyrus and then conveyed to the inferior frontal gyrus for articulation (Hickok & Poeppel, 2004; Price, 2000) and subvocal rehearsal (Buchweitz et al., 2009; Tan et al., 2005). Evidence has shown that children with higher phonological awareness tend to be better at reading in English (Castles & Coltheart, 2004; Goswami & Bryant, 1990; Huang & Hanley, 1995; Wagner & Torgesen, 1987). Better reading in English may rely on access to high quality phonological representations in the temporal cortex and manipulation of those representations in the frontal cortex.

Functional imaging studies have shown that higher connectivity between the left superior temporal gyrus and the left inferior frontal gyrus is correlated with better reading skill among English speaking children and adults (Koyama et al., 2011), and that higher functional connectivity between the superior temporal and inferior frontal gyri is associated with better phonological awareness ability in dyslexic adults (Boets et al., 2013). A study on Chinese-English bilinguals using TBSS found that FA values in the left frontal region and the left lateral sulcus were negatively correlated with the response time of reading English words (Cummine & Boliek, 2013). A DTI study has also found that near posterior superior temporal gyrus, in a cluster at the bend of the AF, higher FA was associated with better phonological awareness ability (Saygin et al., 2013). Together with our study, these results consistently imply that optimal neural transmissions within the direct segment of the left AF provide for better phonological awareness that translates into better English reading skill.

In contrast, along the posterior segment of the left AF, we found that near the superior temporal cortex, FA values from node 6 to node 10 uniquely showed a negative correlation with Chinese reading skill. This negative correlation suggests that better reading performance in Chinese is associated with less engagement of the posterior segment of the left AF. Previous studies found that Chinese reading development was manifested by reduced reliance on phonological processing (i.e., superior temporal gyrus) and increased reliance on visual-orthographic processing (i.e., bilateral occipital-temporal regions) over age (Cao et al., 2010, 2015; X. Liu et al., 2018). Our finding is not surprising, considering that the posterior superior temporal gyrus is thought to be weakly or not engaged in Chinese reading (Xiaojuan Wang et al., 2016). Indeed, the superior temporal part of the left AF was found to contribute significantly more in English than Chinese reading (M. Zhang, Chen, et al., 2014). Phonological awareness was also found to contribute significantly more to English than Chinese reading (Ho et al., 2007; Huang & Hanley, 1995; McBride-Chang, Cho, et al., 2005; Taylor, 2002). We argue that Chinese reading is not positively related to the tissue properties in the posterior superior temporal part of the left AF because phonological processing plays a relatively minor role in Chinese. This argument is also supported by our behavioral data showing that phonological awareness contributed less to Chinese reading (*R*^2^ change = 0.081) than to English reading (*R*^2^ change = 0.227).

We found that the anterior (Figure 1) and posterior (Figure 2) parts of the left AF direct segment showed different types of correlations, with the former being language-common, but the latter being unique to English. Based on previous studies, a specific segment on one tract may play different roles. For example, the FA values of left SLF (nodes 1–19) and right AF (nodes 22–48) were found to be correlated with expressive language, and similar portions (left SLF, nodes 1–27; right AF, nodes 26–47) were also found to be correlated with IQ in typically developing children (Farah et al., 2020). Detailed anatomy of the human AF remains to be seen, so we speculate that the AF may be capable of achieving functional specialization in different nodes by transmitting information from specific cortical regions. We assume the AF may act like a highway with numerous intersections for entry and exit.

### Unique to Chinese Reading

We found that better reading in Chinese, but not in English, was uniquely correlated with FA values along the right AF (nodes 8–48 in the anterior segment; nodes 10–18 and nodes 53–61 in the direct segment), and two clusters of these nodes (nodes 8–48 in the anterior segment; nodes 53–61 in the direct segment) were also related to better visual spatial ability. The importance of visual spatial ability to Chinese reading is also supported by our behavioral data, which showed that visual spatial ability made a unique contribution to Chinese reading but not to English reading. Although the results for the direct segment of the right AF (nodes 53–61) passed only a lenient threshold but not a more stringent threshold of FWE correction, they showed a similar pattern to the results for the right anterior segment of the AF (nodes 8–48). In particular, both clusters of nodes in Figure 3 and Figure 4 located in the right AF were found to be specific to Chinese reading and correlated with visual spatial ability. These results together strengthen the argument that the right AF is important for Chinese reading. Studies have shown that the right AF is strongly associated with visual spatial ability in those with Williams Syndrome (Hoeft et al., 2007) and in typical populations (Barrick et al., 2007; Büchel et al., 2004). Studies have also shown that higher orthographic depth is correlated with a greater role of visual attention in reading (Bavelier et al., 2013; Richlan, 2014; Zhou et al., 2014). Chinese is a deep writing system because of its unsystematic mapping between the components of characters and their pronunciation, so visual spatial processing plays an important role in Chinese reading. In our study, our independent measure of visual spatial ability required children to divide a picture of a holistic pattern into its subcomponents so that blocks could be combined into the holistic pattern. This ability is very useful for the skilled identification of Chinese characters. Most Chinese characters contain a phonetic radical that gives a cue to pronunciation and a semantic radical that gives a cue to meaning, so skilled reading requires the isolation and combination of these subcomponents, so that readers can build the connections from print to pronunciation and meaning (Zhou et al., 2014). In addition, there are many homophones in Chinese. For example, 攻击 (Gōng jī, meaning: attack) versus 公鸡 (Gōng jī, meaning: rooster). These two words share the same pronunciation, but follow strikingly different orthographic structure, so visual spatial information is crucial for skilled reading in Chinese to disambiguate meaning.

A number of functional imaging studies in adults have found greater activation in the right hemisphere in Chinese compared to English readers (Bolger et al., 2005; Tan et al., 2005). In addition, the amplitude of the low frequency fluctuation (ALFF) in the right parietal cortex was found to be correlated with orthographic awareness in Chinese reading (Qian et al., 2016), suggesting that the right hemisphere is important for visual processing of characters. For developing Chinese readers, one study reported that the right parietal showed greater activation in a spelling task compared to a rhyming task, with the former task placing greater demands on visual spatial analysis. In addition, the parietal cortex demonstrated significant developmental increases in activation, suggesting that better Chinese character processing involves more elaborated spatial analysis in the right hemisphere (Cao et al., 2010). All of these functional studies are consistent with our structural study implicating the importance of the right AF in the visual spatial processing of Chinese characters required for skilled reading.

Very few studies have examined the correlation between Chinese reading skill and white matter metrics, and an association in the right AF has not been reported. The studies that have been done focused on the left hemisphere with relatively coarse anatomical specificity. One TBSS study showed that Chinese reading was correlated with radial diffusivity in the left anterior limb of the internal capsule (Qiu et al., 2008). However, a more recent study using spherical deconvolution tractography approach demonstrated that the correlation between cognitive subskills of reading and white matter tracts, including the anterior limb of the internal capsule, may be confounded (Vanderauwera et al., 2015). The AFQ algorithm we implemented in our study was able to investigate behavior correlations with diffusion properties along the bilateral AF, capturing neural information that might only be carried on some specific sections along the tract. Bundles like the AF are relatively large; thus fasciculi might not traverse the whole length but enter and exit at different points. The variation of the correlation between FA and behavioral scores might reflect that a particular segment of the tract has a unique function, in which it communicates the results of local computations to other parts along the tract (Wandell, 2016).

We did not find a correlation between better Chinese tone discrimination ability and the nodes of the right AF that were associated with reading skill in Chinese. Tone discrimination ability was measured by detecting which of four tones were different from the rest, and therefore this measure reflects an individual’s perception of the suprasegmental phonological features. Though studies have suggested greater involvement of the right hemisphere in tone processing (Glasser & Rilling, 2008), it might be communication between the left and the right hemispheres that is crucial for tone processing in Chinese (Ge et al., 2015). Future studies are needed to address this.

To sum up, our study suggests that the direct segment of the left AF is a phonology mediated pathway important for skilled reading in English, whereas the direct and anterior segments of the right AF are visual spatial mediated pathways important for skilled reading in Chinese. The result for English reading is supported by studies showing that children with better phonological awareness ability are more likely to demonstrate better reading skill in English (Goswami & Bryant, 1990; Wagner & Torgesen, 1987). In contrast, better reading in Chinese is not strongly related to the ability to manipulate fine-grained phonological units (Huang & Hanley, 1995; Hu & Catts, 1998; Pan & Chen, 2005), but rather appears to be related to the ability to process the visual spatial information of the characters (McBride-Chang, Chow, et al., 2005; Siok et al., 2009; Sun et al., 2011; L. Y. Yang et al., 2013). Our results suggest that the neural substrate underling visual spatial ability might be a marker of individual differences in Chinese reading skill. However, researchers have argued that visual spatial ability might be a consequence of learning to read. For example, Chinese kindergarteners’ word reading predicted unique variance in visual skill in the first grade (McBride-Chang et al., 2011). Though the relationship between Chinese reading and visual spatial ability is still not clear, our results do show that visual spatial ability is related to reading skill differentially in Chinese versus English.

### Limitations

Although within-subject design avoids intergroup differences such as social, educational, and cultural experiences resulting from a between-subject design, the cross-language comparison in our study might be confounded by whether it is the first (L1) or second (L2) language. For example, L1 and L2 may differ in behavioral proficiency which may affect the underlying processing mechanisms used (Cargnelutti et al., 2019; Sebastian et al., 2011). To control potential confounds, we recruited children who started learning to read both languages at the same time. However, all the subjects learned to speak Chinese earlier and their proficiency in Chinese spoken language was better than in English. It is possible though that the current findings may not reflect a L1 versus L2 effect for the following reasons. First, usually a lower proficiency L2 (here English) engages right hemisphere regions to a greater degree (Dehaene et al., 1997; Sebastian et al., 2011), but we found that the higher proficiency L1 (here Chinese) reading was correlated with the right AF. Second, our finding that the posterior part of the left AF was correlated with English reading is consistent with the majority of previous research on English reading (e.g., Glasser & Rilling, 2008; Saygin et al., 2013). Third, we found Chinese reading was more reliant on visual spatial processing supported by the right hemisphere, whereas English reading was more reliant on phonology processing supported by the left hemisphere. To our knowledge, no previous research has come to a general conclusion that L1 reading is more reliant on visual spatial processing, while L2 reading is more reliant on phonological processing. Nevertheless, further research should investigate a group of English-Chinese simultaneous bilingual children to determine if the effects we demonstrated in sequential bilinguals generalize.

Another limitation is that the underlying white matter property differences in reading across languages found in the current study may be confounded by differences in the task demands of the standard tests used to measure skill in the two languages. Unfortunately, there are no parallel reading skill measures in the two languages. In addition, the large age range (8–12 years) may be another confound. To reduce the effect of the large age range, we controlled for age in data analyses.

### Conclusions

Our study suggests that there are universal neural substrates associated with reading skill in the temporoparietal part of the direct segment in the left AF, indicating a common reliance on mapping between orthographic symbols and their pronunciations. However, there are also language specific mechanisms. Better reading skill in English was correlated with higher fractional anisotropy in the superior temporal part of the direct segment of the left AF, suggesting an important role of phonology in English reading. In contrast, better reading skill in Chinese was correlated with higher fractional anisotropy in the direct and anterior segments of the right AF, suggesting an important role of visual spatial processing in Chinese reading.

## ACKNOWLEDGMENTS

The authors would like to thank all the children and parents who participated in this study. We thank Hojin Jang for his generous help and insights in data analysis coding.

## FUNDING INFORMATION

Li Liu, National Natural Science Foundation of China (https://dx.doi.org/10.13039/501100001809), Award ID: 31970977. Li Liu, National Natural Science Foundation of China (https://dx.doi.org/10.13039/501100001809), Award ID: 31571155. Xiangzhi Meng, National Natural Science Foundation of China (https://dx.doi.org/10.13039/501100001809), Award ID: 31971039. Li Liu, National Basic Research Program of China (973 Program) (https://dx.doi.org/10.13039/501100012166), Award ID: 2014CB846103. Li Liu, Beijing Higher Education Young Elite Teacher Project (https://dx.doi.org/10.13039/501100010026), Award ID: YETP0258. Li Liu, Fundamental Research Funds for the Central Universities (https://dx.doi.org/10.13039/501100012226), Award ID: 2015KJJCB28. Li Liu, The Beijing Brain Initiative of Beijing Municipal Science & Technology Commission, Award ID: Z181100001518003. James Booth, National Institute of Child Health and Human Development (https://dx.doi.org/10.13039/100000071), Award ID: R01 HD042049.

## AUTHOR CONTRIBUTIONS

**Yue Gao**:* Conceptualization; Methodology; Data curation; Formal analysis; Visualization; Writing – original draft. **Xiangzhi Meng**:* Conceptualization; Resources; Funding acquisition; Supervision. **Zilin Bai**:* Methodology; Formal analysis; Validation; Visualization; Writing – review & editing. **Xin Liu**: Conceptualization; Resources. **Manli Zhang**: Data curation; Resources. **Hehui Li**: Data curation; Resources. **Guosheng Ding**: Conceptualization; Resources. **Li Liu**: Conceptualization; Methodology; Writing – original draft; Writing – review & editing; Funding acquisition; Supervision. **James Booth**: Conceptualization; Methodology; Writing – original draft; Writing – review & editing; Funding acquisition; Supervision. (*Equal contribution to this manuscript.)

## TECHNICAL TERMS

Alphabetic language: A type of language that uses letters or a combination of letters to represent speech sound directly.
Phonological awareness: A measurable awareness to detect and manipulate each unit of sounds.
Logographic language: A type of language that uses characters to represent words, mapping onto phonology at syllable level.
White matter: A type of neural tissue of the brain that is made up mainly of myelinated axons, also called *tracts* or *fibers*.
Fractional anisotropy: A value that describes the degree of anisotropy of a diffusion process, and reflects fiber density, axonal diameter, and myelination in white matter.
Tone: An inherent pitch that each syllable in Chinese has, which can change the meaning of a word.
