# Supplementary material for: Left and Right Arcuate Fasciculi Are Uniquely Related to Word Reading Skills in Chinese-English Bilingual Children
Source: Neurobiol Lang (Camb). 2022 Feb 10;3(1):109–31. doi: 10.1162/nol_a_00051 (PMC10158580; doi:10.1162/nol_a_00051)
Supplement: Supplementary file 1 [file nol-3-1-109-s001.pdf]

Supplementary Materials

Table S1. Left AF direct segment: nodes 80-95 specific to English reading skill

Correlations	r	p
Mean FA correlates with visual spatial ability [a]	0.245	0.193
Mean FA correlates with phonological awareness[a]	0.448	0.009

[a] Partialling out sex and age

Table S2. Right AF anterior segment: nodes 8-48 specific to Chinese reading skill

Correlations	r	p
Mean FA correlates with visual spatial ability [a]	0.384	0.048
Mean FA correlates with phonological awareness [a]	-0.167	0.405
Mean FA correlates with tone discrimination [a]	-0.260	0.191

[a] Partialling out sex and age

Table S3. Right AF direct segment: nodes 53-61 specific to Chinese reading skill

Correlations	r	p
Mean FA correlates with visual spatial ability [a]	0.410	0.030
Mean FA correlates with phonological awareness [a]	-0.139	0.482
Mean FA correlates with tone discrimination [a]	0.028	0.887

[a] Partialling out sex and age

Table S4. FA of other tracts that failed to show significant unique correlation with Chinese or English reading skills

Correlations	r-max	p
Left AF anterior - English reading skill [a]	-0.246	0.167
Left AF anterior - Chinese reading skill [b]	-0.324	0.066
Left AF posterior - English reading skill [a]	0.337	0.051
Right AF posterior - English reading skill [a]	0.211	0.230
Right AF posterior - Chinese reading skill [b]	0.346	0.045 [c]

[a] Partialling out Chinese reading skill, sex and age

[b] Partialling out English reading skill, sex and age

[c] Only 1 node passed uncorrected $p<0.05$

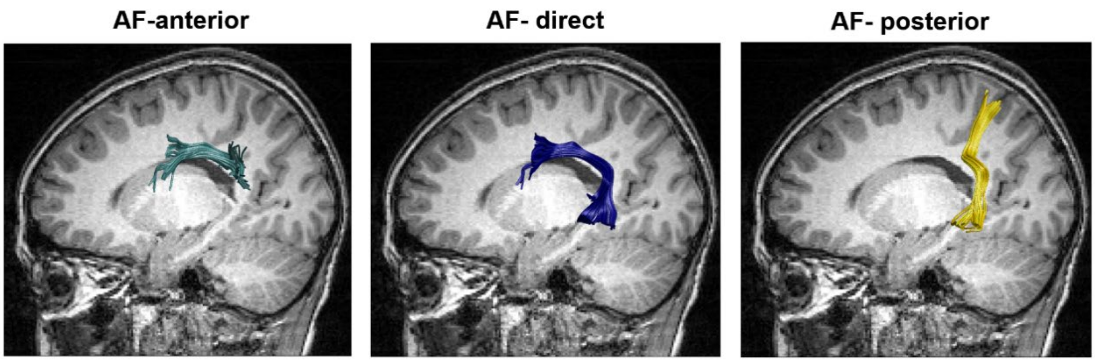

Figure S1. The three tracts (direct, anterior and posterior arcuate fasciculus) depicted on a representative participant

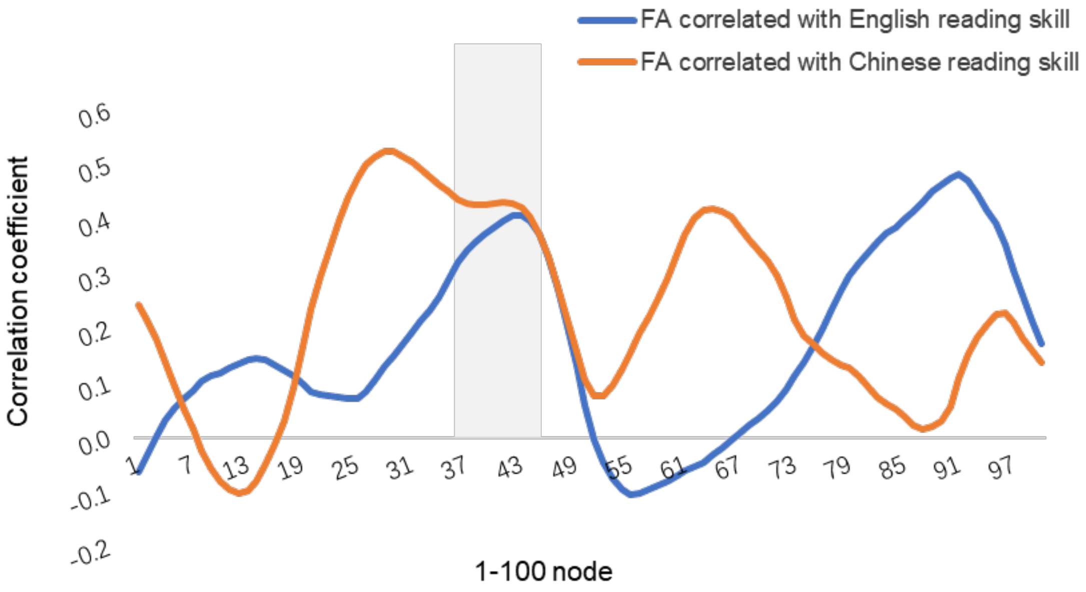

Figure S2. Distribution of correlation coefficients between FA of left AF direct nodes and reading skills in both English and Chinese, partialling sex and age. Nodes 37-45 (in light shadow) were associated with reading skill in both Chinese and English. These results passed a lenient threshold for adjacent nodes ≥ 9 at $p<0.05$ uncorrected, but did not survive a stringent threshold of FWE cluster size correction for adjacent nodes ≥ 16, $p<0.05$). In addition, the mean FA value of these nodes was not correlated with phonological awareness, visual spatial or Chinese tone discrimination ability.

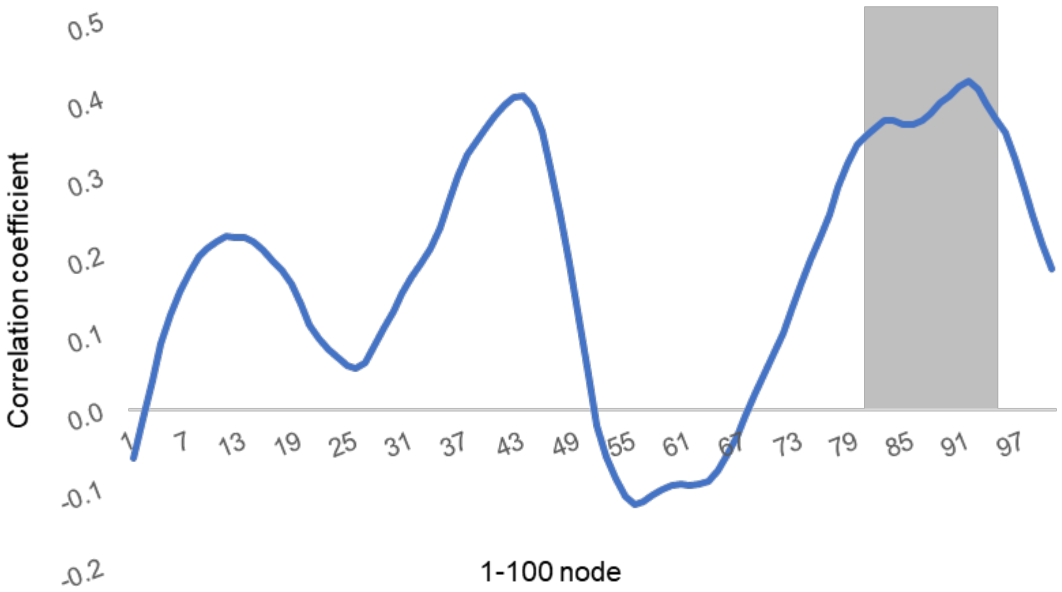

Figure S3. Distribution of correlation coefficients between FA of left AF direct nodes and English reading skill, partialling Chinese reading skill, sex and age. Nodes 80-95 (in dark shadow) were specific to English reading skill, which survived a stringent threshold of FWE cluster size correction for adjacent nodes ≥15, $p<0.05$. In addition, the mean FA value of these nodes was correlated significantly with English phonological awareness, partialling sex and age.

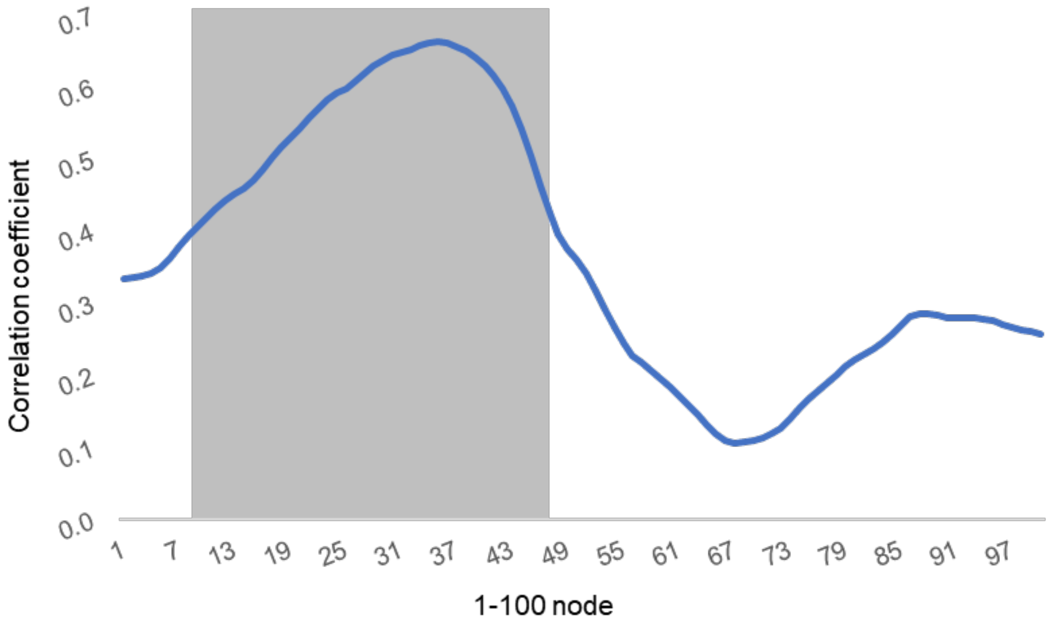

Figure S4. Distribution of correlation coefficients between FA of right AF anterior nodes and Chinese reading skill, partialling English reading, sex and age. Nodes 8-48 (in dark shadow) were specific to Chinese reading skill (survived a stringent threshold of FWE cluster size correction for adjacent nodes ≥ 24, p<0.05). In addition, the mean FA value of these nodes was significantly correlated with visual spatial ability, partialling sex and age.

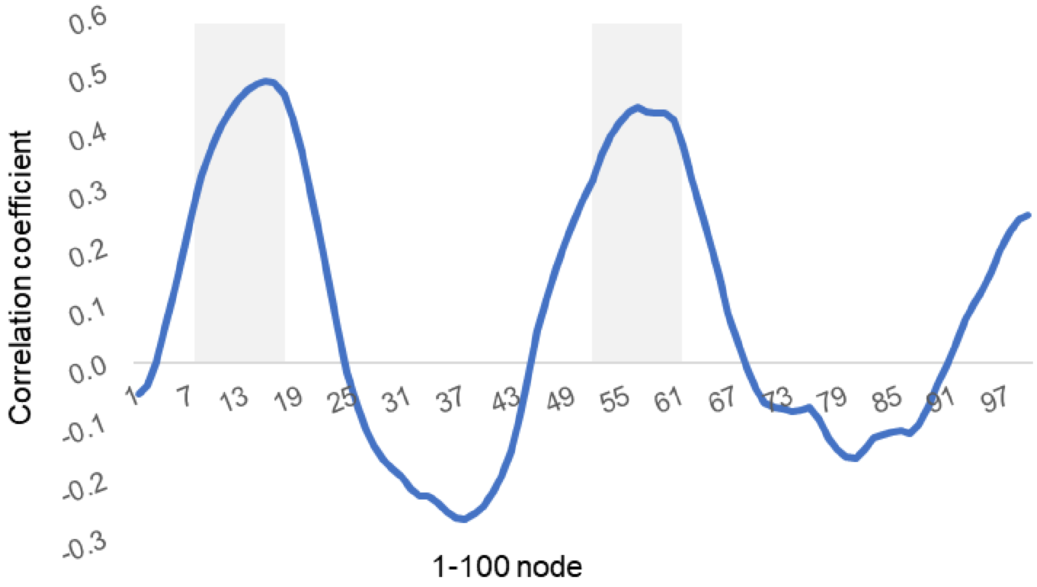

Figure S5. Distribution of correlation coefficients between FA of right AF direct nodes and Chinese reading skill, partialling English reading, sex and age. Nodes 10-18 (in light shadow) and Nodes 53-61(in light shadow) were found to be specific to Chinese reading skill (both passed a lenient threshold for adjacent nodes ≥ 9 at p<0.05 uncorrected, but both clusters did not survive a stringent threshold of FWE cluster size correction for adjacent nodes ≥ 15, p<0.05). Only the mean FA value of the cluster in Nodes 53-61 significantly correlated with visual spatial ability, partialling sex and age.

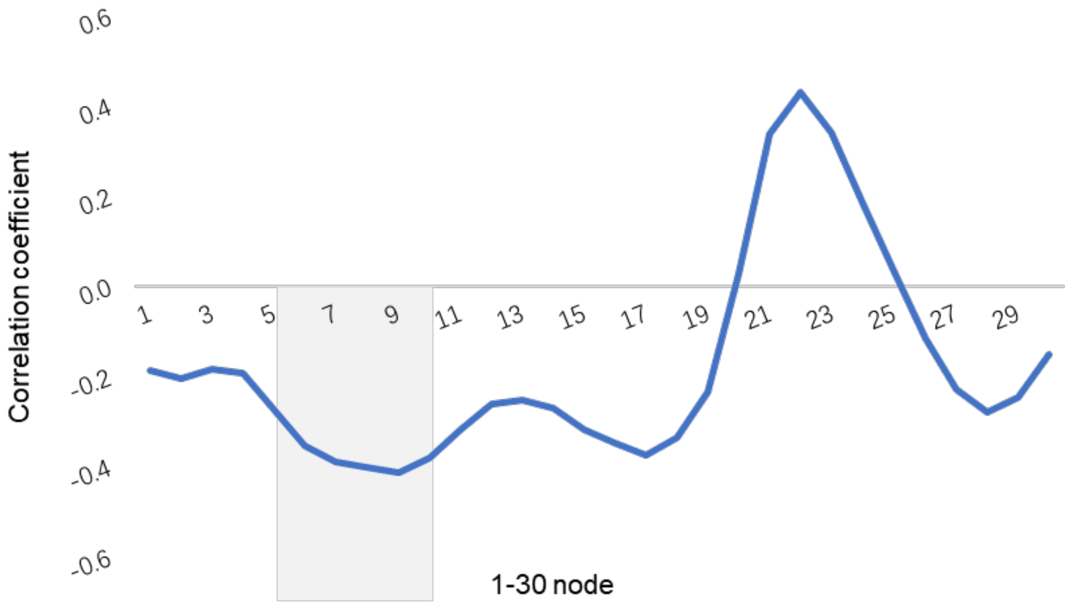

Figure S6. Distribution of correlation coefficients between FA of left AF posterior nodes and Chinese reading skill, partialling English reading, sex and age. Nodes 6-10 (in light shadow) were found to be specific to Chinese reading skill (passed a lenient threshold for adjacent nodes ≥ 3 at $p<0.05$ uncorrected; failed to pass a stringent threshold of FWE cluster size correction for adjacent nodes ≥ 7, $p<0.05$). The mean FA value of the cluster in Nodes 6-10 did not correlate with visual spatial ability, Chinese phonological awareness or Chinese tone discrimination, partialling sex and age.

Detailed description for how AFQ was modified to capture posterior segment of AF

By applying the AFQ codes (https://github.com/YeatmanLab/AFQ) AFQ_Segment_PostArcuate.m and the templates coordinates for left and right posterior AF, we captured the posterior segment of AF. To be specific, "SLFt_roi2_L.mat" was defined as ROI 1 and "L_Parietal.mat" was defined as ROI 2, and tracts going through these two ROIs were defined as left AF_posterior. Thus, by loading the individual WholeBrainFG.mat, dt6.mat and running AFQ_Segment_PostArcuate.m, the bilateral posterior segment of AF was captured.
